# Executive Function: Cognition and Behaviour in Adults with Autism Spectrum Disorders (ASD)

**DOI:** 10.1007/s10803-019-04133-7

**Published:** 2019-07-08

**Authors:** Kate Johnston, Kim Murray, Debbie Spain, Ian Walker, Ailsa Russell

**Affiliations:** 1 Department of Psychology, Institute of Psychiatry, Psychology and Neuroscience, King’s College London, London, UK; 2 South London & Maudsley NHS Foundation Trust, National & Specialist CAMHS, Adolescent At-risk & Forensic Service, Michael Rutter Centre, Maudsley Hospital, De Crespigny Park, London, SE5 8AZ UK; 3 Autism Assessment and Behavioural Genetics Clinic, South London and Maudsley Foundation NHS Trust, London, UK; 4 MRC Centre for Social Genetic and Developmental Psychiatry, King’s College London, London, SE5 8AF UK; 5 Department of Psychology, University of Bath, Bath, BA2 7AY UK; 6 Centre for Applied Autism Research, Department of Psychology, University of Bath, Bath, BA2 7AY UK

**Keywords:** Autism spectrum disorder, Executive function, Adult, Dysexecutive syndrome, Neuropsychology

## Abstract

Studies of executive function (EF) in autism spectrum disorder (ASD) have reported mixed findings. Possible confounds include EF domain assessed and co-occurring neurodevelopmental diagnoses. EF task performance across multiple domains and everyday function of autistic adults (n = 110) was significantly different to age- and IQ-matched controls (n = 31). Although significantly more likely to fall into the clinically impaired range, 35.8% of the ASD group showed no impairment on EF measures. Factor analysis revealed a single unifying EF construct rather than a selective pattern of impairment. Dysexecutive behaviours were frequently reported in the ASD group, unrelated to Autism symptoms, EF task performance or co-occurring conditions. This study suggests autistic adults can experience clinically significant executive function difficulties and co-occuring dysexecutive behaviours that are disabling in everyday life.

## Introduction

Executive function is a complex cognitive construct incorporating a number of processes associated with higher-level thought and behaviour which develop across the lifespan (e.g. Diamond and Goldman-Rakic 1989). Thought to be essential to goal oriented behaviour, executive functions are closely related to other cognitive processes such as memory and attention and are also important in affective processing and behaviour. There are several cognitive models of executive function (EF) (e.g. Norman and Shallice 1986; Stuss and Benson 1984; Baddeley 1996; Rolls 1996; Shallice and Burgess 1991a, b). Some suggest that EF can be explained by a unitary ‘central executive’ (Baddeley 1996) whereas others suggest a range of individual but associated processes which operate in parallel without an overarching control system (Goldman-Rakic 1995). Clinically, these models have been translated into various cognitive assessment tools designed to tap specific processes such as concept formation and cognitive flexibility e.g. the Wisconsin Card Sorting test (Berg 1948), Brixton Test (Burgess and Shallice 1997) whilst others are designed to assess overarching processes such as organisation/planning e.g. Six elements test (Shallice and Burgess 1991a). Executive functioning has also been a key area of interest in a variety of psychiatric and neurodevelopmental conditions.

Autism spectrum disorder (ASD) is a neurodevelopmental condition characterised by impairments in social communication and a restricted, repetitive and stereotyped pattern of interests and behaviours (APA 2013). Impairments in EF are widely cited in ASD and executive dysfunction has been posited to underlie the core difficulties (e.g. Russell 1997; Ozonoff et al. 1991). For example, Lopez et al. (2005) found cognitive flexibility, working memory and inhibition, but not fluency and planning, were strongly associated with stereotyped and repetitive behaviours in adults with ASD. Using multiple regression analyses, the authors found that no single EF predicted ASD symptomatology but rather that a model including both relative cognitive strengths (working memory, inhibition) and weaknesses (flexibility, fluency) had the highest predictive power. Hill and Bird (2006) also found an association between executive dysfunction and ASD symptomatology. Findings from brain imaging studies also lend support to the idea that executive function difficulties can be seen as central to ASD and may relate to neuroimaging findings of structural and functional differences in the prefrontal cortex (PFC) (e.g. Gilbert et al. 2009; Luna et al. 2002; Castelli et al. 2002). There is some evidence that the association between EF and ASD core symptoms may be less than straightforward. A study by Jones et al. (2018) found that performance on EF tasks was not uniquely associated with social communication or repetitive behaviour measures, but rather was indirectly related through performance on Theory of Mind tasks.

A number of issues regarding investigations into the nature of EF impairments in ASD are relevant. Firstly, executive functioning has been found to be impaired across a number of psychiatric and developmental disorders (e.g. Hosenbocus and Chahal 2012; Goodkind et al. 2015). Attention deficit hyperactivity disorder (ADHD) is similarly associated with executive function impairments, particularly response inhibition. ADHD is the most commonly reported co-occurring condition in children and adults with ASD, with more than 40% of adults in a recent study also meeting diagnostic criteria for ADHD (Joshi et al. 2013). Disentangling the clinical and neuropsychological phenomenology of these 2 distinct but highly related developmental conditions (Gillberg 2010) is less than straightforward. A study of attention in adults, particularly response inhibition, found an ASD group’s performance could be characterised as slow and accurate while an ADHD group were consistent with an impulsive style of responding i.e. fast with errors. However, both groups were impaired relative to neurotypical controls (Johnston et al. 2013).

Secondly, impairment in performance on EF tasks may be a consequence of slowed processing speed. Several studies report difficulties in timed tasks of executive function due to difficulties with initiation and psychomotor speed (Hill and Bird 2006) and a subsequently slow and accurate response style in individuals with ASD (Johnston et al. 2011; Hill and Bird 2006). Spek et al. (2009) report that speed of processing accounted for poor phonemic fluency in a group of adults with HFA rather than difficulties with strategy formation or shifting. There have been findings of greater difficulties on ‘open-ended’ rather than structured EF tasks for an ASD group in comparison with typically developing controls (e.g. Van Eylen et al. 2015).

Nonetheless, there are consistent findings of EF impairment in ASD across child, adolescent and adult samples but inconsistent findings in respect of the domain of function(s) that is impaired. It is possible that the mixed findings can be accounted for by study design in respect of the heterogeneity of the population studied (e.g. age and IQ), presence of co-occurring mental health and other neurodevelopmental conditions as well as measurement paradigm. Demetriou et al. (2018) carried out a meta-analysis of 235 studies (comprising 14,081 participants; 6816 with ASD) investigating sub-domains of EF in ASD. They stratified the analysis by age to account for developmental maturation in brain function and considered a number of potential moderators of EF impairment including gender, sample and control group characteristics, IQ differences, and modality of test administration amongst others. No differential pattern of EF impairment was found when studies were combined and moderate effect sizes for all EF sub-domains were observed, which the authors identified as concept formation, mental flexibility, fluency, planning, response inhibition and working memory. Informant and self-report of behavioural and functional facets of EF impairment were found to show the highest amount of heterogeneity, contributing to larger effect sizes than experimental and psychometric tasks. Effects of the proposed moderators were not significant and the authors concluded a broad pattern of EF impairment across the domains identified, with no selective pattern of impairment. Informant based questionnaires had greater clinical utility in terms of distinguishing between Autistic and non-autistic controls and the authors concluded that such questionnaires may have greater ecological validity. Of note, studies included in this review did not exclude participants with comorbid ADHD or other psychiatric diagnoses and so the impact of these upon executive function is unclear.

Behaviours suggestive of executive problems such as perseveration, rigidity and difficulty self-monitoring have been examined using the Dysexecutive Questionnaire (DEX—Wilson et al. 1996) with relative impairments in DEX self- and informant-report scores compared with non-ASD controls and scores in line with data from brain injured samples for an ASD adult group (Hill and Bird 2006; Cederlund et al. 2010). Differences between parent completed DEX of adolescents with Asperger’s syndrome and a typically developing control group have also been reported (Channon et al. 2001). The behavior rating inventory of executive function (BRIEF) (Gioia et al. 2000) has been used in several studies of young people with ASD, with a diverse pattern of impairments relative to non-ASD controls reported across the different domains e.g. in metacognitive skills and flexibility (Rosenthal et al. 2013); and planning and flexibility (Van den Bergh et al. 2014). The latter study identified no relationships between BRIEF scores and ASD symptoms. A single study (Wallace et al. 2016) reports adult ASD data from the BRIEF finding impairments in both flexibility and metacognition and these impairments in ‘everyday’ executive functioning were associated with anxiety and depression respectively. Similar confounds arise when conceptualising the measurement of behavioural aspects of EF impairment in ASD as do with assessment of cognitive facets i.e. co-occurring mental health and/or neurodevelopmental conditions, age, IQ and theory of mind difficulties. For example, the Behavioural Regulation Index of the BRIEF comprises three sub-scales or indices; inhibition, shift and emotional control. The contribution of ADHD symptoms and core ASD characteristics to these dimensions may be significant; Gioia et al. (2000) found elevated scores on each domain of the BRIEF in children with ADHD and this finding has also been replicated in adolescents with ADHD (Toplak et al. 2008). Similar findings have been reported in adults with ADHD using a self-rated measure of EF difficulties with ‘behavioural’ EF difficulties being more strongly associated with everyday functioning than standard cognitive EF assessments (Barkley and Murphy 2010).

In summary, it remains unclear whether impairments in EF are truly characteristic of ASD in relation to both cognition and behaviour given that previous studies have not used rigorous diagnostic processes for commonly co-occurring disorders known to affect executive functioning (such as ADHD). This means it is difficult to draw conclusions as to a selective, autism-specific pattern of impairment. In the present study, we aim to investigate performance across several important cognitive domains of executive function; planning, generativity, set shifting and strategy formation and related behaviour in adults with a clinical diagnosis of ASD, homogeneity in respect of ASD diagnostic pathway, intellectual ability in the average range while taking account of co-occurring clinical disorders and processing speed. The study will be restricted to adults to control for the developmental confounds in respect of brain maturation and executive function.

Research question:

1. To investigate the presence and pattern of EF performance, across multiple domains of EF measurement, in adults with ASD without co-occuring ADHD.

Hypotheses:

1. The ASD group will have significantly slower processing speed on cognitive tasks, as measured by the time taken to complete tasks.
2. There will be a significant association between cognitive and behavioural measures of EF.

## Method

### Participants

Participants with ASD were recruited from a specialist diagnostic service for adults. This was a clinical sample of consecutive participants meeting inclusion criteria over a period of 3 years, all of whom completed the same neuropsychological and diagnostic battery and consented to take part in the study All participants received a clinical diagnosis of ASD made by a consultant psychiatrist and nurse specialist and met ICD-10 criteria for Autism (Autism; n = 26), Asperger’s syndrome (AS; n = 53), atypical autism (n = 21) or pervasive developmental disorder not otherwise specified (PDD-NOS; n = 10). Where a reliable informant-based developmental history was available the autism diagnostic interview-revised (ADI-R; Lord et al. 1994) was completed (n = 78) and in cases where this was not available then participants underwent the autism diagnostic observation schedule (ADOS; Lord et al. 1989; n = 48). In a small number of cases for clinical soundness, both the ADI-R and the ADOS were completed (n = 19) with both measures showing agreement. In three cases where one or both measures were sub-threshold on one domain, diagnoses of PDD-NOS, Asperger’s syndrome and atypical autism were given. All participants received their diagnosis in adulthood. Mean ASD group scores on the ADI-R were as follows [ADI-R Communication Domain Mean Score 10.08 (SD = 5.11); ADI-R reciprocal social interaction domain mean score = 12.47 (SD = 6.01); ADI-R repetitive behaviour domain mean score = 3.58 (SD = 2.03)]. Of the ASD group completing the ADI-R, 25 (32%) of individuals scored below diagnostic threshold on the communication domain, 22 (28%) scored below threshold on the RSI domain and 25 (32%) on the RB domain. Where ADI-R scores were below diagnostic threshold, diagnosis was supplemented with the ADOS-G (n = 5) with above threshold scores for autism spectrum disorder. Twenty-one (19%) of the sample received a diagnosis of atypical autism in keeping with below threshold scores on one domain of the ADI-R. Five individuals scored below diagnostic threshold on > 1 domain on the ADI-R and ADOS-G scores were not available. Of those completing the ADOS-G (n = 48, 43.6%), mean ADOS communication and social interaction summary score = 11.10 (SD = 4.09).

Psychiatric co-morbidity was assessed via clinical interview and recorded as ICD-10 diagnosis on the case file with the exception of attention deficit hyperactivity disorder (ADHD), which was made according to DSM-IV criteria as per National Institute for Health and Care Excellence (NICE) guidance.

Exclusion criteria were: Verbal IQ < 80 (given the lack of normative data for EF measures for people with low IQ), any other neurodevelopmental disorder diagnosis such as attention deficit hyperactivity disorder (ADHD), Fragile X syndrome and/or Velocardio facial syndrome, any prior brain injury, diagnosis of psychosis, substance misuse, and/or an eating disorder.

The Control group was recruited from a volunteer database of individuals willing to participate in research, and an opportunity sample. The two groups were matched by age, gender and verbal IQ (see Table 1). The study had ethical approval from the local ethics committee. Participants were provided with information about the study and asked to provide written consent to participate if they agreed.

**Table 1:** Mean age, Verbal IQ and gender distribution across the ASD and Control groups

Group (N)	Age *M* (SD)	Range	Sex% (F:M)	VIQ *M* (SD)	Range
ASD (110)	33.35 (10.86)	46	19:81	105.13 (13.49)	59
Control group (31)	30.84 (7.3)	24	26:84	102.66 (13.78)	45

### Sample Size

There was no formal sample size calculation. However, as we expected relatively high communality across the sub-domains of EF, a sample size of 100 was considered appropriate as per guidance (MacCallum et al. 1999).

### Measures

The following measures were used to assess intellectual ability and executive functioning. All measures were part of a clinical neuropsychological test battery used as part of a wider autism diagnostic assessment and have been previously used in studies with autistic adults. Tests were chosen to have high ecological validity given that this should allow for the best association with everyday living skills.

#### General Ability: The Wechsler Adult Intelligence Scale—Third Edition (WAIS-III; Weschler 1997)

An empirically derived seven subtest short form of the WAIS-III was used to assess intellectual ability (for more information see Axelrod et al. 2001).

#### Planning: The Behavioural Assessment of Dysexecutive Syndrome (Wilson et al. 1996)

The BADS is an ecologically valid measure of EF (Norris and Tate 2000) and consists of six subtests in total. The following subtests were used in the present study.

#### The Key Search

This task assesses strategy formation and participants have to indicate how they would search a field (represented by a square box on a piece of paper) to make absolutely certain that they would find their keys if they had misplaced them in the field. The strategies are scored according to their efficiency, effectiveness and time taken to complete the search.

#### The Zoo Map

This task comprises of two subtests, one assessing spontaneous planning and the other assessing planning within a structured context. The former requires participants to plan a route around the zoo visiting predefined locations while adhering to a number of rules. In the latter, participants have to visit locations in the zoo in a predefined order. The initial planning time, total planning time, accuracy and error rates are recorded.

#### Inhibition and Cognitive Flexibility: The Hayling and Brixton Test (Burgess and Shallice 1997)

The Hayling test assesses both verbal initiation and verbal inhibition in the form of correct and incorrect sentence completion, respectively. Timing scores are collected for both and error rates are collected for the inhibition subsection. The Brixton task is a measure of cognitive flexibility (analogous to the Wisconsin Card Sorting Task) and involves predicting the sequence of a blue circle moving around ten potential positions. The pattern changes unexpectedly and individuals have to identify the new pattern to predict the blue circles next correct location. The number of errors is scored.

#### Generativity/Fluency: Controlled Oral Word Association (COWA; Spreen and Strauss 1991)

The COWA or FAS was used to measure verbal fluency. This test involves producing as many words beginning with the letter ‘F’ in 1 min as possible. This is then repeated with the letters ‘A’ and ‘S’. The total number of correct words is scored along with the total number of repetitions and rule-breaks.

#### Time

A variable to account for speed on tasks was calculated by summing time taken in seconds on several EF tasks where time is recorded but specific limits are not enforced (Zoo Map 1 time taken + Zoo Map 2 time taken + Key Search time taken + Hayling total time). This measure of speed/time was chosen after consultation with a statistician who recommended this as an effective way to compare the speed at which groups completed timed but open ended tasks. Rather than processing speed, this was designed simply to assess speed of task completion.

#### Behavioural Characteristics: The DEX Questionnaire (Wilson et al. 1996)

The DEX questionnaire consists of 20 items rated on a 5 point Likert scale pertaining to difficulties associated with dysexecutive syndrome. The self and informant- based versions were used in the present study. Three sub-scales or factors within the DEX have been empirically derived: Behavioural, Cognitive and Emotional difficulties.

#### ASD symptoms: The Autism Quotient Questionnaire (Baron-Cohen et al. 2001)

The AQ is a well-validated screening measure for ASD traits and was used in the Control group. No control participants scored above the suggested ASD cut-off of 32 (Woodberry-Smith et al. 2005) and so none were excluded on this basis.

### Procedure

All participants were tested in a quiet room by an assistant psychologist trained in test administration and working under the supervision of a qualified clinical psychologist.

### Data Analysis Strategy

Data were initially analysed to examine between group differences in mean scores on the main variables. The proportion of individuals meeting criteria for clinical abnormality on each domain of executive function was also compared across groups. To investigate the hypothesised variance in performance across executive function tests, exploratory factor analysis was conducted on the summary test scores of the main 5 EF measures. A single component was extracted which allowed a single variable ‘EF function’ to be created. This variable was entered into regression equations to examine its contribution to the variance in self-report behavioural measures of executive difficulties and scores on autism measures.

## Results

The groups did not differ in respect of mean age, verbal IQ and were matched in terms of gender distribution (see Table 1).

Of the ASD group, 48 (43.6%) did not meet ICD-10 criteria for co-occurring mental health diagnoses. Twenty-one (19%) of participants in the ASD group met diagnostic criteria for OCD, 25 (22%) met ICD-10 criteria for depression, 10 (9%) for Generalised Anxiety Disorder (GAD), 17 (15.4%) for social anxiety disorder, 8 (7.2%) for Agoraphobia and 3 (2.7%) met criteria for a personality disorder. Participants were grouped into those with and without a co-occurring psychiatric diagnosis and a between groups analysis conducted across all measures of executive function and IQ. Analysis showed that there were only two significant group differences between individuals with ASD and a co-occurring anxiety, mood or personality disorder and those with ASD alone across all of the variables of interest: Zoo Map 1 raw (t_(105)_ = 2.03, p < 0.05) and sequence score (t_(106)_ = 2.02, p < 0.05) but not at reduced α (0.01) to correct for multiple comparisons. Therefore individuals with OCD, anxiety, mood and personality disorder were included in the final analysis.

### EF Task Mean Scores

Table 2 shows the median and interquartile range for each group across the test battery. Mann–Whitney tests were used to compare group differences due to non-normal data distribution on the main EF measures. T-tests were used to consider group differences on variables that were normally distributed. Table 2 shows that there were significant between group differences across a range of EF tasks. Large group differences were particularly seen on measures of verbal generativity (FAS), verbal initiation and inhibition (Hayling) and cognitive flexibility (Brixton).

**Table 2:** Scores across each measure of executive function across groups

Test	ASD sample Mdn (IQR)	Control sample Mdn (IQR)	Z
BADS			
Zoo Map Profile Score	2 (2)	3 (2)	− 2.41*
Zoo Map 1 time	176 (143)	146 (83)	NS
Zoo Map 1 sequence	4 (6)	7 (4)	− 2.26*
Zoo Map 1 errors	1 (3)	1 (2)	NS
Zoo Map 2 time	77 (47)	48 (37)	− 3.40**
Zoo Map 2 sequence	8 (0)	0 (0)	NS
Zoo Map 2 errors	0 (0)	0 (0)	− 2.08*
Key Search profile score	3 (4)	4 (1)	− 2.05*
FAS			
Total correct	32.5 (17)	42 (13)	− 3.99**
HAYLING (H)			
H1 Standard Score	5 (2)	6 (0)	− 3.84**
H2 Standard Score	6 (2)	6 (0)	− 3.12**
H2 Error Standard Score	6 (1)	7 (1)	− 3.51**
Total H Standard Score	6 (2)	6 (1)	− 4.78**
BRIXTON			
Standard Score	6 (3.75)	7 (3)	− 3.11**

*< 0.05, **< 0.01

### Clinically Significant Impairment

**Fig. 1:**
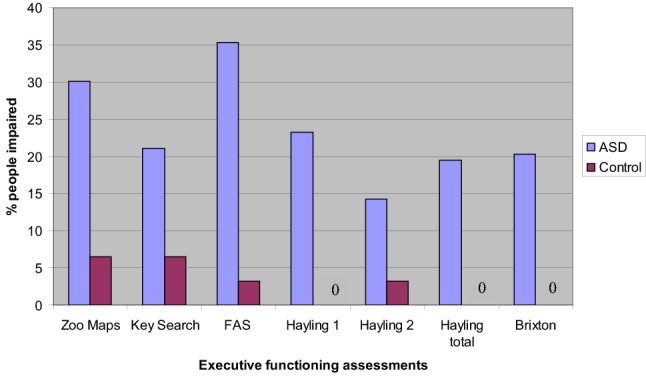
Percentage of control and ASD participants falling in the clinically impaired range (< 5th percentile)

Scores below the 5th percentile in the majority of neuropsychological tasks are generally taken as indicative of clinically meaningful impairment and have been used in similar studies (e.g. Hill and Bird 2006). Figure 1 shows the percentage of people falling within the clinically impaired range on each of the EF tasks according to published test norms. Chi squared analysis was used to compare groups; significantly greater numbers of participants in the ASD group scored in the clinically impaired range on the FAS χ_(1, N = 141)_^2^ = 11.86 (p < 0.001) and Hayling 1 χ_(2, N = 141)_^2^ = 9.41 (p < 0.01) tests. The Hayling 2 χ_(2, N = 141)_^2^ = 4.73 (p < 0.05), Hayling Total χ_(2, N = 141)_^2^ = 7.35 (p < 0.05), Zoo Map χ_(1, N = 141)_^2^ = 5.96 (p < 0.05) and Brixton χ_(2, N = 141)_^2^ = 7.35 (p < 0.05) tests were significant but not at reduced α (0.01) to correct for multiple comparisons.

To address the possibility that impairment might be heavily loaded on a few individuals within the ASD group, analyses were conducted to examine whether the groups differed in distribution of clinical impairment (see Table 3) (χ_(5, N = 141)_^2^ = 24.32, p < .0001). Of note, more than one-third of the ASD group were not in the clinically impaired range on any of the EF tasks.

**Table 3:** Number of tasks in the impaired range by group

Number of EF tasks	Number of people scoring in the impaired range in the ASD group (%)	Number of people scoring in the impaired range in the Control group (%)
5	2 (1.8)	0
4	5 (4.6)	0
3	6 (5.5)	0
2	24 (22.0)	0
1	33 (30.3)	5 (16.1%)
0	39 (35.8%)	26 (83.9%)

In order to examine possible timing differences on the measures of executive function between the two groups, a ‘time’ variable was created by adding the time in seconds taken by each participant to complete all timed tasks. The mean ‘time’ score for participants with ASD differed significantly from that of the Control group, ASD mean task time = 500.6 s (range 119–1518 s) and Control group mean task time = 365.3 s (range 166–946 s); t_(75.4)_ = − 3.64, p < .001, 95% confidence interval of the mean difference lower = − 215.7, upper = − 55.27.

### Behavioural Characteristics

Table 4 shows the results of self- and informant-report DEX questionnaires. On both self and informant versions, the ASD group was reported to have significantly greater difficulties on all sub-scales of the DEX measure. There is a large amount of missing data for the DEX due to administrative issues unrelated to participants.

**Table 4:** Mean self and informant ratings on the DEX questionnaire across the ASD and Control groups

	Mean self-rating (SD)			Mean informant rating (SD)		
	ASD (n = 65)	Control (n = 29)	t (df)	ASD (n = 52)	Control (n = 19)	T (df)
Behaviour	13.20 (6.99)	8.10 (4.72)	− 3.57 (92)***	17.63 (6.63)	5.21 (4.58)	− 8.77 (66)***
Cognition	9.22 (3.83)	4.48 (2.91)	− 5.92 (91)***	9.83 (4.07)	2.21 (2.34)	− 9.81 (70)***
Emotion	6.34 (2.64)	3.58 (1.97)	− 2.76 (91)***	7.21 (2.59)	2.63 (2.06)	− 6.93 (69)***
Total Dex score	28.98 (11.48)	16.17 (8.46)	− 5.36 (89)***	35.24 (11.97)	10.05 (8.23)	− 9.74 (63)***

***p < .001

The standardisation data for the DEX suggested that the informant scale can be taken as a more reliable indicator of difficulties in individuals with reduced insight. Paired samples t-tests suggested that a significant difference between self and informant ratings was evident in both the ASD and the Control group albeit in opposing directions; ASD group mean self-informant discrepancy = − 4.99 (SD = 13.65), t = − 2.45, df = 43, p < .05; Control group mean self-informant discrepancy = 6.44 (SD = 9.001), t = 3.13, p < .01.

Normative data for self-report for individuals with brain injury on the DEX is 27.21 (SD = 14.48) and informant-rated 32.85 (SD = 15.98). In the present study, the group with ASD and their informants report levels of characteristics associated with dysexecutive syndrome similar to those reported by people with acquired brain injury.

Further exploration of participants who had completed the DEX questionnaire with co-morbid psychiatric diagnosis information available in the clinic report revealed no significant differences between the mean scores for the ASD group with a co-morbid psychiatric diagnosis (n = 30) and those without a co-morbid diagnosis (n = 31) (ASD co-morbid group mean DEX total self-rating score = 30.37 (SD = 11.15); ASD non co-morbid group mean DEX total self-rating score = 27.22 (SD = 11.70); t = − 1.076, df = 59, p = .286). The same non-significant finding was also present when DEX informant ratings were compared between the co-morbid and non-co-morbid groups.

### Factor Analysis

All 5 main EF measures (Zoo Map and Key Search total profile scores, total number of correct words on the FAS, Hayling overall total score and Brixton scaled score) were significantly correlated with each other (see Table 5).

**Table 5:** Non-parametric correlations (Spearman’s rho) between the summary scores on the 5 main executive function measures (n = 141)

Measure		1	2	3	4	5
1. Zoo Map profile score	Correlation coefficient	1.000				
	Sig. (2-tailed)	–				
2. Key Search profile score	Correlation coefficient	.371**	1.000			
	Sig. (2-tailed)	.000	–			
3. FAS total words correct	Correlation coefficient	.202*	.207*	1.000		
	Sig. (2-tailed)	.016	.014	–		
4. Hayling overall total scaled score	Correlation coefficient	.312**	.434**	.439**	1.000	
	Sig. (2-tailed)	.000	.000	.000	–	
5. Brixton error scaled score	Correlation coefficient	.327**	.330**	.251**	.375**	1.000
	Sig. (2-tailed)	.000	.000	.003	.000	–

*p < 0.05, **p < 0.01 ***p < 0.001

Exploratory factor analysis using principal components analysis was performed to consider factors underlying performance on the 5 domains of EF function for all participants. Initial Eigen values indicated that the 1st component explained 46% of the variance. The remaining 4 components had Eigen values < 1.0 and thus just 1 factor was extracted from the data and although equamax rotation was specified, the single factor solution could not be rotated. This single factor was used to produce a regression factor score, creating a variable labelled ‘EF function’. The single factor underlying the 5 EF tests is consistent with Baddeley’s (1974) construct of a single overarching executive function.

**Fig. 2:**
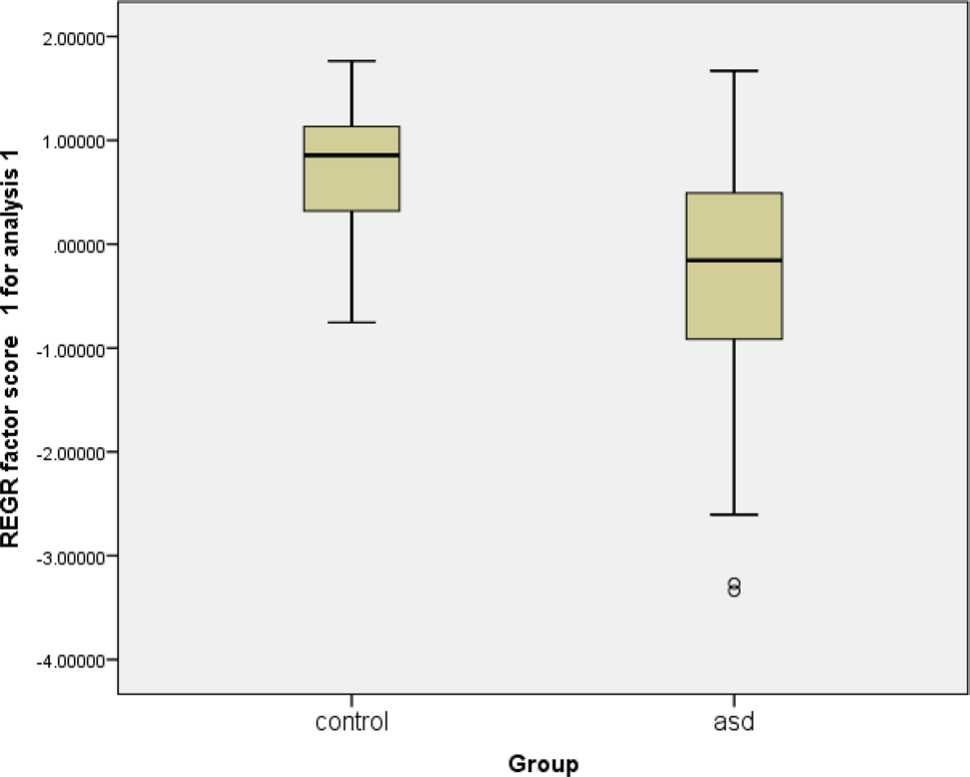
‘EF function’ score by group

The ASD and Control groups differed significantly in terms of mean scores on ‘EF function’ (see Fig. 2). (ASD mean EF score = − .20734 (SD = 0.993), Control group mean EF score = 0.72235 (SD = 0.114); (equal variances not assumed Levine’s f = 6.758, p = .01); t = 6.331, df = 78.457, p < .0001, 95% CI of the difference 0.6373–1.222).

To further consider the role ASD symptoms might contribute to executive difficulties, ADI-R scores, number of co-occurring psychiatric conditions, age, Verbal IQ and the ‘time’ variable were entered into a regression equation. The model was significant (F = 27.12, p < .0001, R^2^ = .739) with Verbal IQ being a positive predictor of EF and time being a negative predictor of EF. None of the other variables made a significant contribution.

As already noted, a clinical presentation characteristic of executive function difficulties as measured by the DEX rating scales was prevalent in the ASD group. Considering the variables that might contribute to the variance in the behavioural, cognitive and emotional features rated on the DEX, multiple linear regression was used. The ‘EF function’ score, time score, ADI communication, reciprocal social interaction and repetitive behaviour scores were entered into the equation. The model was non-significant F = .971, p = .458; with R^2^ = .139 suggesting that neither performance on neuropsychological tests of executive function, speed of processing nor ASD symptoms contributed to the DEX self-report total rating score. A non-significant finding was similarly found when the dependent variable was specified as the DEX informant report total score.

## Discussion

The aims of the present study were to investigate the pattern of performance of adults with ASD across a range of cognitive tests of executive function and a questionnaire measure of everyday dysexecutive symptoms and to compare these to age and IQ matched controls and remove the potential confound of co-occurring ADHD. We were interested to understand the pattern of performance in autistic individuals without ADHD given the potential impact of this on EF. We hypothesised that autistic adults would show a slow performance style across timed measures. Finally, we hypothesised that a high level of impairment on cognitive tasks of EF would be accompanied by self- and informant-reports of dysexecutive difficulties impacting upon everyday function.

### Patterns of EF Performance in Adults with ASD

In relation to our broad research question, investigating the presence and pattern of EF performance, across multiple domains of EF measurement we found that adults with ASD had lower scores relative to matched controls across measures of planning (Zoo Map, Key Search), generativity (Hayling test, verbal fluency) and flexibility (Brixton). A significantly greater proportion of the ASD group than controls had scores in the ‘clinically abnormal’ range on a measure of generativity (35% impaired) and a measure of response initiation (24%). These measures require generation of a verbal response, with the former less scaffolded than the latter. Clinical impairment was widespread in the ASD group, 20–30% had clinically impaired scores on measures of planning, 20% on a measure of flexibility with fewer impaired on a measure of verbal inhibition (15%). The results of a factor analysis across all participants indicated a single factor underlying performance on the neuropsychological domains measured in this study and the ASD group differed significantly from the Control group on this factor.

#### Hypothesis 1

The ASD group will have significantly slower processing speed on cognitive tasks, as measured by the time taken to complete tasks.

In support of hypothesis 1, adults with ASD took significantly longer to complete EF tasks. Since both groups were matched for IQ and age this slow performance style appears to be consistent with previous reports (e.g. Johnston et al. 2011; Hill and Bird 2006) noting a slow and accurate response style amongst individuals with ASD.

#### Hypothesis 2

There will be a significant association between cognitive and behavioural measures of EF.

Individuals with ASD reported high levels of dysexecutive difficulties on the DEX questionnaire. However, scores on the behavioural measure of dysexecutive syndrome did not bear close relation to performance on tests of executive function, speed of task performance or to measures of ASD symptoms and thus our hypothesis was not supported.

It is important to note that one-third of the group with ASD were not in the clinically impaired range on any of the EF tasks which is at odds with other similar studies (e.g. Hill and Bird 2006). This may reflect differences in the way that clinical impairment was calculated in our study (using normative data from test manuals) and a larger sample size. The exclusion of participants with co-occurring ADHD may also be relevant to this finding, particularly the relatively low rates of impairment on a test of inhibition.

In order to exclude the possibility that these results are explained by a small number of individuals performing poorly on EF tasks we examined the number of individuals who had scores in the clinical impairment range across 0, 1, 2, 3, 4 or all 5 EF measures. Results showed that whilst none of the control sample were impaired on more than one measure, a third of participants with ASD were impaired at two or more tests. Interestingly very few individuals with ASD were impaired across all measures suggesting variance within executive functioning as might be expected given such a heterogeneous range of tasks assessed and the variability within the autism spectrum. This is consistent with the results of a recent meta-analysis (Demetriou et al. 2018) where combining findings from a wide range of studies highlighted a broad spectrum of EF impairment in ASD.

Consistent with our hypothesis that adults with ASD would show slow task completion is the finding that the most common EF impairment in the ASD group was on a task of verbal fluency (FAS) which is considered a measure of processing speed as well as a measure of generativity (Spek et al. 2009). Score attainment on this verbal fluency task is time-sensitive. There was also a significant difference between groups on the time taken to complete the Zoo Map 2 task. We calculated an overall timing score on the basis of timed, open-ended tasks i.e. where time taken did not influence performance scores. Taken together, these findings suggest that on both open-ended and time-constrained neuropsychological measures, the ASD group were significantly slower. Our ‘Time’ variable was significantly negatively associated with the overall ‘EF’ factor, suggesting that slow and effortful processing was not supportive of more accurate task performance.

In relation to dysexecutive difficulties, informants rated these as being somewhat higher than the individuals themselves in the ASD group. This is consistent with normative data for the measure. However the converse discrepancy between self- and informant-ratings was present in the Control group and thus it is hard to draw conclusions. Self- and informant-rated scores in the ASD group on the behavioural measure of dysexecutive difficulties were very similar to those reported by individuals with acquired brain injury. In the brain injury literature, performance on more traditional tests of neuropsychological function have not always been found to align with functional difficulties (Shallice and Burgess 1991a, b). We used more ecologically valid measures of EF in the present study to try and overcome this methodological issue. Previous studies with children have shown a strong association between the BRIEF and core ASD symptoms (Gilotty et al. 2002) and also failed to find an association between everyday executive function and ASD symptoms in children (van den Bergh et al. 2014). Our findings are consistent with those of Wilson et al. (2014) who reported a similar lack of association between impaired performance on cognitive assessments of EF and ASD symptoms in adults. Curiously, the timing variable while relevant to EF task performance did not account for variance in dysexecutive behaviour scores. Furthermore, our findings do not appear to reflect a lack of ‘insight’ into dysexecutive problems on the part of autistic adults as the informant-rated DEX scores also showed no association with ASD symptoms. Further investigation of the items on the DEX questionnaire is warranted to elicit a greater understanding of the phenomenology and potential underpinnings of high scores by adults with ASD. Although not directly related to ASD symptoms as measured in this study, the items potentially are not related to neurocognitive indices either. Whilst these are clinically interesting findings, an important caveat is the large amount of missing data for the DEX this is in part due to administrative processes rather than issues related to participants. These findings should therefore be interpreted with caution as this is not necessarily a representative sample of adults with ASD.

Our findings showed that individuals in the ASD group were impaired across measures with both implicit and explicit expectations. For instance, 20% of our sample fell in the clinical abnormality range on the Brixton task which has clear instructions that are much more explicit than other measures of cognitive flexibility such as the widely used Wisconsin Card Sorting Test (WCST). Similarly, the Zoo Map test is considered a more ‘constrained’ measure of planning and is less often impaired in individuals with ASD (White 2013), but our findings indicated that it was on this test that the ASD group showed the second highest level of impairment. On Zoo Map 2 which is a more structured task, participants with ASD made relatively more errors and performed more slowly than control participants although these differences did not reach significance. Interestingly the more open-ended task (Key Search) was the only one where participants with ASD did not show statistically significantly different rates of clinical abnormality as compared to age and IQ matched controls.

### Limitations of the Current Study

The findings of the present study and conclusions that can be drawn are limited by the relatively narrow and blunt measure of executive functions utilised in our clinical sample. We used well validated and standardised clinical neuropsychological measures of executive function to investigate our hypotheses. Although this brings advantages in respect of ecological validity, the complex and inter-related nature of executive processes means precise conclusions relating to cognitive models of EF cannot be drawn from these broad measures. Moreover, the clinic diagnostic algorithm meant ADI-R and ADOS scores were not available for all participants uniformly, and these analyses are limited in this respect. Relatedly, the use of older assessment tools (ADOS-G and WAIS-III) in the current study reflects the length of time over which this data was collected. Studies comparing these tools with their current counterparts have generally shown high rates of agreement (e.g. correlation coefficients ranging from .83 to .94 for WAIS-III and WAIS-IV; Wechsler 2008) or there have been no significant changes (e.g. ADOS-G to ADOS-2, module 4) meaning that the current findings are relevant to current criteria/practice. Forthcoming changes to the classification of autism in the ICD-11 will be relevant in understanding the nature and pattern of both executive functioning and everyday living skills in autism given the discontinuity in diagnostic criteria. It is suggested that a significant proportion of individuals who currently meet diagnostic thresholds will no longer do so under ICD-11 (or DSM-5) criteria (e.g. Doernberg and Hollander 2016; Wilson et al. 2013). This raises important issues about the generalisability of existing research in this area and access to clinical/educational services.

### Future Research and Conclusions

The results of the current study do bring some clarity to the literature on EF function in ASD. In studying adults without intellectual disability and with robust diagnostic assessment of both ASD and relevant co-morbidity, particularly excluding ADHD, important and relevant issues are highlighted for people with ASD and clinicians. Individuals with ASD reported high levels of dysexecutive symptoms that were functionally impairing in everyday life across behavioural, cognitive and emotional domains. A considerable proportion of the adults with ASD in this study were impaired across one or more neuropsychological measures of executive function. Generativity and response initiation were most affected in respect of clinical impairment. These data present preliminary evidence that performance on EF measures appears to reflect a single underlying construct. Functional and neurocognitive indices of EF impairment were independent of ASD symptoms and of each other. These findings suggest that EF is an additional important co-occurring condition to consider in ASD. Of equal importance is the finding that one-third of adults were not in the impaired range on any of the neurocognitive measures. Future research seeking to more precisely understand the nature of EF in ASD should seek to delineate assessment paradigms further by reducing the influences of processing speed, theory of mind ability and implicit task demands on task performance. It would also be useful to explore differences in executive functioning between adults with both ASD and ADHD, ASD only and controls to further understand the pattern of EF strengths and difficulties and the relative contribution made by each neurodevelopmental condition. Future investigation into the nature of everyday ‘dysexecutive’ behaviours is warranted. At a clinical level, it would seem beneficial to ensure that EF assessment is multi-method and that impairments are conceptualised as (a) potentially significant in the formulation of clinical problems and (b) important to scaffold when modifying psychosocial interventions to meet the needs of autistic people.
